# An introduction to sample preparation and imaging by cryo-electron microscopy for structural biology

**DOI:** 10.1016/j.ymeth.2016.02.017

**Published:** 2016-05-01

**Authors:** Rebecca F. Thompson, Matt Walker, C. Alistair Siebert, Stephen P. Muench, Neil A. Ranson

**Affiliations:** a Astbury Centre for Structural Molecular Biology, University of Leeds, Leeds LS2 9JT, United Kingdom; b MLW Consulting, 11 Race Hill, Launceston, Cornwall PL15 9BB, United Kingdom; c Electron Bio-Imaging Centre, Harwell Science and Innovation Campus, Didcot, Oxfordshire OX11 0DE, United Kingdom

**Keywords:** Electron microscopy

## Abstract

Transmission electron microscopy (EM) is a versatile technique that can be used to image biological specimens ranging from intact eukaryotic cells to individual proteins >150 kDa. There are several strategies for preparing samples for imaging by EM, including negative staining and cryogenic freezing. In the last few years, cryo-EM has undergone a ‘resolution revolution’, owing to both advances in imaging hardware, image processing software, and improvements in sample preparation, leading to growing number of researchers using cryo-EM as a research tool. However, cryo-EM is still a rapidly growing field, with unique challenges. Here, we summarise considerations for imaging of a range of specimens from macromolecular complexes to cells using EM.

## 1 Introduction

Transmission electron microscopy (EM) can be used to provide structural information on a range of biological specimens from cells to macromolecules. The resolution of the information that can be acquired using EM is dependent on the properties of the sample, the specimen preparation method used, the technical specification of the electron microscope, and imaging parameters. 3D information can be obtained from the 2D EM data collected in a variety of ways, including single particle analysis [1], [2], helical reconstruction [3], electron tomography (with or without subtomogram averaging) [4], [5], and 2D crystallography [6], [7]. These methods of data processing are suitable for different samples; single particle analysis for purified, ‘homogenous’ protein complexes (such as the ribosome [8]), helical reconstruction for protein assemblies with helical symmetry (such as microtubules [9]), tomography for ‘unique’ assemblies (such as organelles and cells [10]), and 2D crystallography for proteins, significantly smaller than 150 kDa that form ordered 2D arrays (such as bacteriorhodopsin [11]). Single particle analysis uses multiple, untilted 2D projection images that contain many ‘single particles’ with different angular orientations. The angular relationships between each view of the specimen can be calculated in the images to provide 3D information. Electron tomography and 2D crystallography obtain different angular views by tilting the same specimen many times (typically tilting the specimen between ±65° and taking an image every 2°, yielding 65 images of the same area). The angular relationship between each image is known, because the tilt increment is defined, and so this can be used to reconstruct 3D information. The generation of 3D structural information from 2D micrographs and electron diffraction patterns using the processing methods above has been reviewed extensively elsewhere [1], [2], [6], [7], [12].

**Fig. 1:**
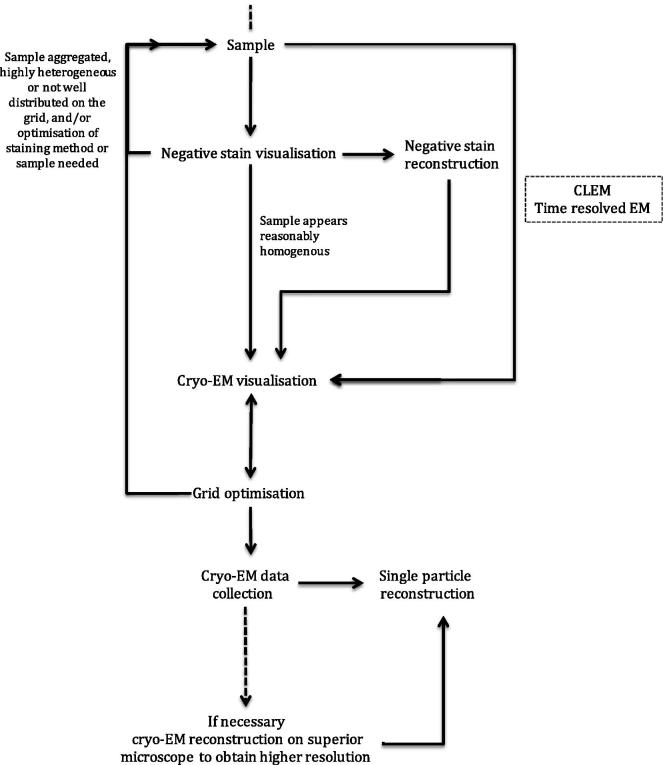
Example workflow for structure determination by single particle EM.

Here, steps towards biological structure determination by EM are discussed with a focus on sample preparation and imaging of specimens for single particle analysis (example workflow shown in Fig. 1) and electron tomography, although many of the concepts and considerations discussed are transferrable.

## 2 Sample preparation

When generating a specimen for imaging by EM, the ultimate specimen preparation technique may influence considerations at the sample preparation or purification stage (Table 1). To perform single particle EM, thousands of identical particles are imaged and computationally averaged together. The ideal single-particle specimen is thus as homogenous as possible. Heterogeneity can result from both conformational variation and flexibility, or by compositional changes, such as the presence or absence of different subunits, or binding partners. Compositional variation can be reduced biochemically using appropriate protein purification methods, although a biochemically ‘pure’ sample does not ensure the sample will appear homogenous in the EM; structural arrangements and intrinsic flexibility may produce a broad range of conformations. In the case of conformational variations, and in some cases of compositional heterogeneity, solution buffer conditions can dramatically alter the appearance of a sample in the microscope; high-throughput screening can be used to identify optimal buffer compositions [13]. Chemical crosslinking may also be a useful tool for reducing sample heterogeneity; methods such as GRAFIX have been developed that permit the purification of a sample based on stoichiometry (Reviewed in [14]), however chemical crosslinking may introduce artefacts such as trapping the complex in a non-native or non-functional form. Other approaches to reducing conformational flexibility include ligand-induced stabilisation, where molecules such as a substrate, ligand, inhibitor or protein/nucleic acid binding partners can promote stable complex formation, a strategy that is well established in X-ray crystallography [15].

A commonly encountered problem for some specimens is obtaining sufficient protein concentrations to prepare EM grids. While the total volume required to make a grid is small (∼3–5 μl), achieving sufficiently high concentrations on an EM grid can be challenging. These issues are specific to the protein/specimen being produced, and generally are overcome in the ‘wet lab’. However, some strategies during EM sample preparation can be used to increase particle density, such as treatment of the grid surface (Section 3.3).

Although optimising the sample before attempting to image it remains vital, improved quality of data from direct electron detecting cameras, and novel imaging processing algorithms mean some heterogeneity in the dataset can be dealt with during the processing stages [16]. Indeed, sorting out this heterogeneity, brought about by the functional cycle of the protein of interest, can provide invaluable insights into biological mechanisms [8], [17].

## 3 Grid preparation

Once the specimen is ready, it must be prepared for imaging by EM. To image with electrons, a microscope is maintained under a high vacuum, in which hydrated biological specimens would dehydrate rapidly. Thus to image a biological specimen, it must be fixed, preferably in a native (or native-like) state. Another consideration is sample thickness. In some cases, the sample may be too thick for electrons to be ‘transmitted’, a prerequisite for transmission EM, and so the specimen may need to be processed to make it thinner. The choice of preparation technique is ultimately determined by the nature of the sample and the resolution required for the intended biological insight, but common methods include negative staining, plastic embedding/sectioning, and vitrification.

EM grids are traditionally 3.05 mm across, and made from a mesh of metal such as copper, gold, nickel, molybdenum or rhodium. Copper grids are most commonly used, but gold supports are becoming more common, especially when cells are to be grown directly onto the grid, as they are non-toxic to cells. The metal mesh supports the film on top. The mesh size, or number of squares across the grid, is defined as the number of squares in one inch. For example, a 200-mesh grid has 20 squares across in each direction, and a 300 mesh 30 squares. 200–400 mesh grids are most commonly used for cryo-EM. Over the metal support a support film is deposited, different support films are used for different purposes (Section 3.3). Finer mesh sizes provide more support for the film, but when the grid is tilted, the bars can block the beam. Therefore 200 mesh grids are most commonly used for tilt series data collection.

### 3.1 Negative staining

*Typical usage: The high contrast and speed of grid preparation of negative stained samples makes it ideal for assessing sample purity, concentration, heterogeneity and conformational flexibility. Low-resolution 3D reconstructions from stained data can also provide starting models for higher resolution cryo-EM studies*.

*Advantages: High speed of sample preparation and good specimen contrast. Additionally, there is the ability to label with antibodies or gold to further aid functional studies and determine stoichiometry. Grid can be easily kept for many years and re-imaged*.

*Disadvantages: Structures limited to modest resolution (∼>20 Å), surface topology only, possibility of staining artefacts, incompatible with some buffers and/or reagents*.

**Fig. 2:**
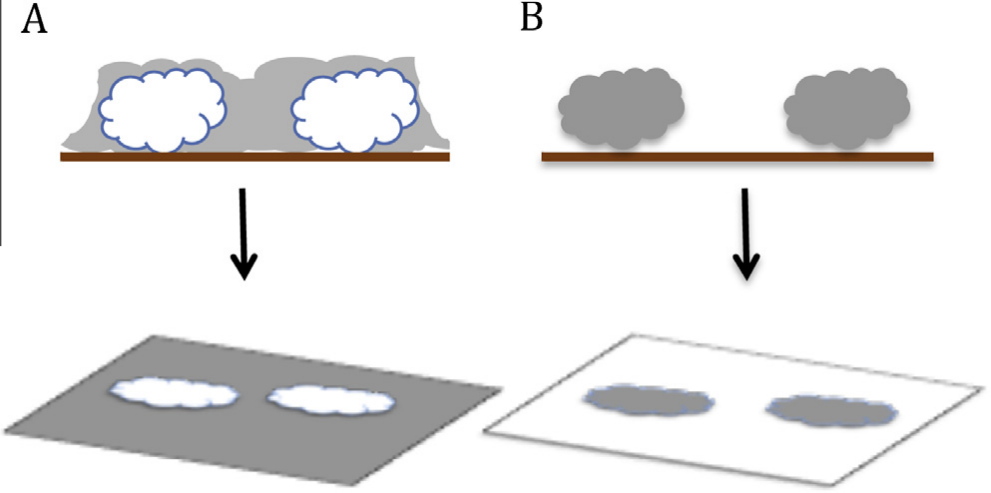
Positive and negative staining using heavy metal salts. In negative staining (A) the stain fully envelops the macromolecular complex; in the micrograph the complex appears white on a dark background. Positive staining (B) results in a small amount of stain forming a thin shell around the molecule, meaning the sample appears dark against a light background.

Negative stain is a common method for examining a specimen, especially macromolecular complexes, at room temperature. Many variations of the negative staining technique have been documented since the late 1950s, using several different heavy metal stains [18]. Typically, specimens are adsorbed onto a thin (∼10 nm) continuous carbon support film that has been rendered hydrophilic (Section 3.3). It is then stained with a solution of heavy metal salt, commonly 1–2% (w/v) uranyl acetate or uranyl formate and blotted to ensure a thin layer of stain, with no stain migrating to the back side of the grid. Some common buffer components (Table 1) are known to adversely affect the quality of staining. However, a large dilution of a concentrated specimen with a compatible buffer immediately prior to application to the grid is the easiest way to ameliorate this. If this is not possible, washing the grid prior to staining is also an option. The staining process quickly dehydrates the specimen and envelops it in stain (where the sample is visualised by the absence of stain, negative staining occurs; where the sample itself becomes stained, positive staining occurs) (Fig. 2). The resulting shell of heavy metal atoms generates amplitude contrast, and a relatively high signal to noise (SNR) ratio (image formation in TEM has been reviewed in [12]). The correct stain depth must be achieved for optimal imaging, with information being lost when the stain is too deep or too shallow. The dehydration of the specimen and its deformation during adsorption are drawbacks of negative stain sample preparation. Additionally, the resolution of stained images is limited by the grain size of the stain, which determines how well the stain envelope reflects the structure of the object [19]. The use of a continuous carbon support film can result in samples adopting a preferred orientation on the grid, complicating 3D structure determination due to a lack of views. Despite these problems, negative stained samples can generate 3D data and provide invaluable biological insight [20], [21]. For example, negative staining can be used to elucidate the binding of small molecule inhibitors in a matter of weeks [22], assess conformational changes [23], [24], complex stability [25] and subunit stoichiometry/position [26], [27]. The process of negative staining has been reviewed fully in [28].

Negative staining can also be combined with vitrification (Section 3.2), in a sample preparation technique known as cryo-negative staining. This method combines the high contrast of using a heavy metal stain with the protective effects of cryogenic preservation (Reviewed in [29]).

### 3.2 Vitrification

**Fig. 3:**
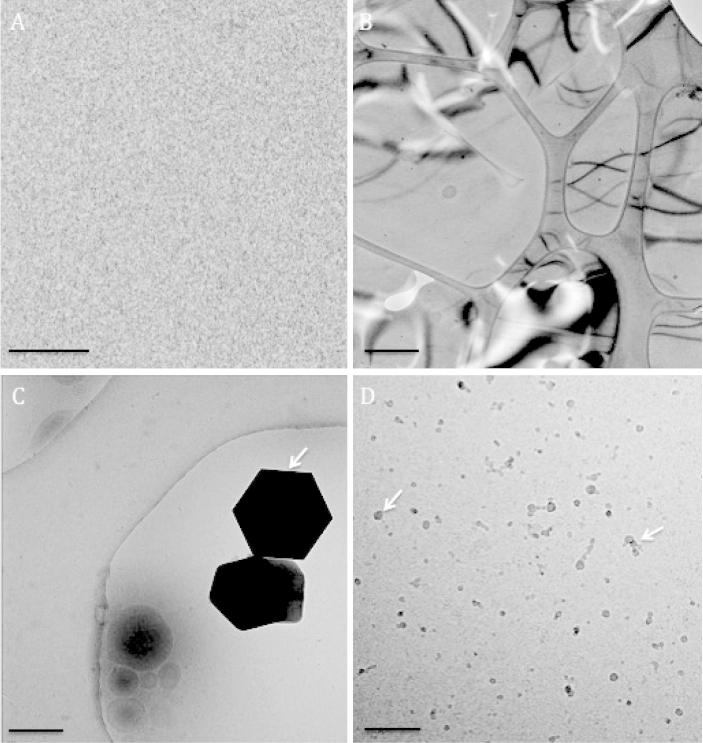
Examples of vitreous and non-vitreous ice. A) Empty, vitreous ice (Scale bar 50 nm). B) Hexagonal ice (scale bar 400 nm). C) Large ice crystal (white arrow) (scale bar 400 nm). D) Probable ethane contamination (scale bar 200 nm).

Sample preparation techniques such as staining and plastic embedding involve the dehydration of biological specimens, which fundamentally removes them from their native, aqueous environment. Cryo-immobilisation preserves that aqueous environment, whist also ameliorating radiation damage [30]. Imaging of cryogenically immobilised samples by EM is known as cryo-EM. The sample must be frozen extremely rapidly, at a rate of ∼10^6^ °C/s, so that the water in, and surrounding the specimen is fixed in a vitreous state [31]. If freezing occurs too slowly, or the specimen is subsequently warmed above −137 °C, the temperature at which water devitrifies [31], crystalline ice is formed. In our experience, warming the sample more than ∼−160 °C can give poor quality ice. Ice contamination compromises the structural integrity of the specimen, as crystals withdraw water molecules from the hydration shells of the specimen, or the specimen itself (Fig. 3). Formation of crystalline ice also degrades image quality as they diffract electrons. Contamination can also occur during the freezing process, such as hexagonal ice, which can be reduced by working in humidity controlled environments and minimising ice contamination in the liquid nitrogen.

Although cryo-EM has been in development since the 1970s, the large number of high-resolution structures deposited in the last two years has promoted increased interest in the technique [32], [33], [34], [35], [36]. Here, cryo-immobilisation methods are discussed.

#### 3.2.1 Plunge freezing

*Typical usage: A wide range of samples, from small (∼150 kDa) macromolecules to whole eukaryotic cells can be vitrified and imaged by cryo-EM*.

*Advantages: Sample is visualised in a native-like state. Processing of data can yield high-resolution, 3D structural information*.

*Disadvantages: Biological samples must be imaged with low electron doses to prevent radiation damage resulting in poor contrast. It is sometimes non-trivial to optimise sample distribution and ice thickness*.

**Fig. 4:**
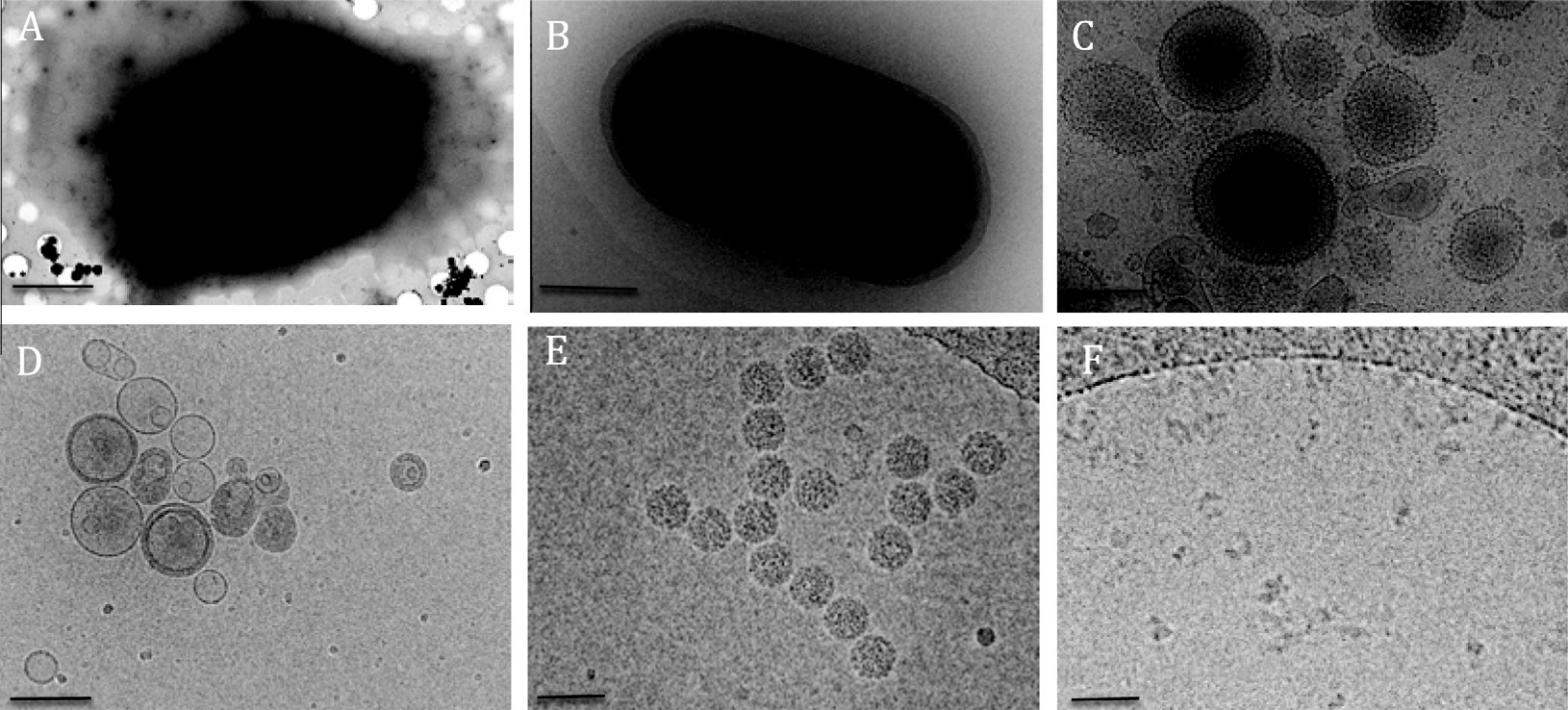
Multi-scale imaging by cryo-EM. A) Eukaryotic cells (scale bar 6 μm). B) Prokaryotic cells (scale bar 0.5 μm). C) Isolated organelles, in this case microsomes (scale bar 200 nm). D) Synthetic liposomes (scale bar 100 nm) E) Viruses (scale bar 50 nm). F) Macromolecular complexes (scale bar 25 nm).

For thin specimens such as suspensions of macromolecules, 2D or very small 3D crystals, small cells, and the thin edge of larger cells, cryo-immobilisation can be achieved by blotting to generate a thin film of liquid containing the sample, and then rapidly freezing it by plunging into a cryogen (Fig. 4). The most widely used cryogens are liquid ethane or propane, cooled by liquid nitrogen [37]. Plunge freezing thin samples is used for a wide range of specimens. For each, the ice layer should be as thin as possible without distorting the specimen and whilst fully embedding the particle, to maximise sample contrast. Failure to fully embed the specimen can lead to preferential radiation damage of the specimen outside of the ice layer. Blotting and plunging can be achieved with simple devices, often built in-house [38]. However, these devices often struggle to provide consistent results within and between batches of grids prepared. Several commercial instruments are available that achieve more reproducible results, including models by FEI, Leica and Gatan (Table 2). Commercially available freezing apparatus offer different features that may be beneficial when working with certain samples. For example, single-sided blotting may be advantageous when preparing grids of adherent cells. Double sided blotting can result in the stripping of some cells from the grid and onto the blotting paper. Single sided blotting can be performed from the ‘back side’ of the gird, where cells are not adhered, circumventing the problems caused by double sided blotting. Additionally, some macromolecular complexes may benefit from a low (<5 °C) temperature and/or humidity control in the freezing chamber. These considerations may influence the choice of freezing apparatus, where a choice is available.

In many facilities, wide ranges of samples are prepared using the same plunge-freezing instrument. Care should be taken to decontaminate the device appropriately after use, for example with 70% (v/v) ethanol. For samples that represent a biohazard, plunge freezers can be placed in a biosafety cabinet. This protects the user against aerosols, and enables the device to be fumigated, enhancing biosafety.

#### 3.2.2 High pressure freezing

*Typical usage: Thick specimens such as the nuclear region of eukaryotic cells, tissue sections, and whole organisms such as Caenorhabditis elegans that cannot be prepared by plunge freezing*.

*Advantages: Sample is preserved in a native-like state. Can result in high-resolution 3D structural information*.

*Disadvantages: To be visualised by TEM, i.e. become electron transparent, the sample must be sectioned or thinned by focused ion beam (FIB) milling after freezing. There is the potential to generate artefacts in this process, and it can be technically challenging*.

While samples thinner than the edge of a large cells may be vitrified by plunge freezing, thicker samples (<1 μm) such as the nuclear and perinuclear regions of eukaryotic cells, or tissue sections are likely to experience some crystalline ice formation during plunge freezing. For these specimens, high-pressure freezing (HPF) is an effective alternative. HPF involves raising the pressure of the sample to ∼2000 bar while dropping the temperature using liquid nitrogen [39]. For samples >100 μm thick up to ∼300 μm thick they can be vitrified using HPF, but must be subsequently sectioned to be thin enough for TEM. Sectioning can be performed under cryogenic conditions, which gives the best preservation of the sample. This technique, known as cryo-electron microscopy of vitreous sections (CEMOVIS), uses cryo-ultramicrotomy with a diamond knife to produce sections 40–100 nm thick [40], [41], [42]. It is a useful technique, capable of imaging thick specimens in a native-like state, but it is technically extremely challenging. A common alternative approach is to perform freeze substitution on the sample, which enables sectioning and imaging at room temperature [43], [44]. Freeze substitution involves gradually warming the specimen and replacing the water with acetone. The sample can then be stained, embedded in resin and sectioned. Freeze substitution introduces fewer artefacts than traditional plastic embedding, but small ice crystals do form causing rearrangement of cell structures, and staining is not uniform so interpreting images can be challenging.

An alternative to sectioning is the use of FIB milling to reduce specimen thickness [45], [46]. FIB milling is carried out on frozen hydrated specimens in a dual beam scanning EM/FIB instrument [47]. A focused beam of ions is rastered across the surface of the sample, removing surface atoms in a process known as sputtering. The scanning electron microscope (SEM) allows simultaneous, non-destructive monitoring of the milling process [47]. Gallium ions are commonly used because of their volatility and low melting point [47]. The ion beam is generated by a liquid metal ion source and liberated by an extraction electrode. Electromagnetic lenses and apertures are used to focus the ion beam, and deflectors to control the pattern of milling. FIB milling can introduce artefacts, as sputtered material can redeposit on the surface of the specimen, although an appropriately positioned, cooled anti-contaminator device can reduce this. Differential milling rates can also produce streaking or curtaining across the surface, and local heating/devitrification can also occur. However conditions have been established where temperatures do not rise enough for this to be a routine problem [48]. Specific steps can be taken to ensure a smooth surface during milling, such as use of a gas injection system to create a organometallic platinum layer covering the specimen, that protects it from some artefacts caused by irregular sputtering during milling [49]. FIB milling can either be used in conjunction with imaging by SEM, as in serial block-face imaging, or the milled sample transferred to a TEM [45], [46]. Since the first successful cryo-FIB experiment in 2003 [50], workflows have been developed to improve and optimise the technique including *in situ* cryo-lamella preparation of cells grown on EM grids. Cryo-FIB technology is still developing; one particularly exciting development is the implementation of correlative light microscopy in combination with FIB milling [51].

### 3.3 Support films

A key consideration in grid preparation is the choice of grid and support film. For cryo-EM perforated carbon films are generally used, allowing the specimen to be imaged in ice suspended between the holes in the carbon support film. Continuous carbon films are used for negative staining. However, in our experience samples can have dramatically differing affinities for carbon films. The surface properties of the carbon can be altered by a variety of processes, including exposure to UV radiation, glow discharge, poly-l-lysine or detergent treatment [52], [53]. In cryo-EM, altering the charge properties of the carbon film can change the partitioning of the sample into the holes, but this needs to be optimised for each sample. Some samples have a very high affinity for the carbon film, these samples sometimes benefit from a thin, continuous carbon film layered over the perforated film. Such a carbon layer can improve particle distribution, but needs to be thin (<10 nm) to prevent adding excessive noise to the images. Such thin carbon films can also be extremely fragile, so there is a trade off between carbon stability and thickness. Perforated amorphous carbon films are available commercially, and can consist of regular arrays of equal sized holes, as with Quantifoil® and C-Flat™ grids, or irregular, as in lacey carbon. Perforated carbon film grids with an ultrathin (3–5 nm) continuous carbon film can also be purchased commercially, or made in-house.

Amorphous carbon support films are widely used, but are not without their problems. They can be inconsistent between batches, and the quality of the amorphous carbon film can deteriorate over time. Additionally, instability of amorphous carbon films contributes to beam-induced particle motion, blurring the image of the specimen [54]. Novel materials are being developed to tackle these problems, including gold support films, graphene and doped silicone carbide films, all of which appear to reduce beam induced particle movement [55], [56], [57]. Improvements to support films have the potential to significantly increase the quality of both non-tilt and tilted cryo-EM data collection.

## 4 Imaging

As shown in Table 1, the optimal hardware for EM is highly dependent on the imaging experiment planned. There is often a trade off between performance, cost, convenience and the availability of instrument time. Here, the choices of electron source, electron detector, and hardware to boost SNR in images are discussed.

### 4.1 Electron source

The electron gun of an electron microscope extracts and accelerates electrons and is typically either a thermionic electron source or a field assisted thermionic emitter such as a Schottky emission gun, commonly known as a field emission gun (FEG) [58]. Common conventional thermionic sources include tungsten filaments or lanthanum hexaboride crystals (LaB6), which are heated so the voltage potential exceeds the work function required to liberate electrons, and operate at voltages between 80 and 200 kV [58]. By comparison, a FEG is an extremely fine tungsten filament coated with zirconium oxide, typically operated at extraction voltages of 200–300 kV, and at 1800 K (1526 °C) [58]. FEGs are much brighter and more coherent compared with conventional thermionic sources, and so are preferred for high-resolution EM studies. However they are significantly more expensive both to purchase and maintain. The choice of gun should therefore reflect the experiment to be carried out. For example negative stain EM does not require the brightness and coherence of a FEG source.

### 4.2 Electron detectors

The high-energy electrons used in TEM imaging are recorded using a detector. Recording devices include photographic film, charged coupled device (CCD) cameras or direct electron detectors (DED). Cryo-EM images of biological specimens are intrinsically noisy due to the low electron doses used to prevent specimen radiation damage. A perfect detector would add no noise but in practice, all detectors do. This can be expressed as the detective quantum efficiency (DQE) of the detector, the square of the output signal to noise ratio (SNR_o_) over the input signal to noise ratio (SNR_i_) [59]. A perfect detector would have a DQE of 1 across all spatial frequencies.

$$\mathrm{DQE}={\mathrm{SNR}}_{o}^{2}/{\mathrm{SNR}}_{i}^{2}$$

Historically, film has was used for EM data collection due to its large field of view and extremely small “pixel” (the grain size of silver halide crystals in the emulsion). However, recording large data sets using film is inconvenient and time consuming. Film emulsion contains water and introduces contamination into the column of the electron microscope, and thus requires desiccation before loading and anti-contamination devices within the microscope. This also limited the number of images which could be collected in a days data collection to ∼100. Film must also be developed and digitised using an extremely accurate and thus expensive densitometer. CCD cameras were developed as a more convenient solution, which also enabled the development of automated microscope alignment and image acquisition procedures, as well as significantly improving the ability to collect a tilt series. CCDs utilise a phosphor (or similar) scintillator, which induces the emission of photons when electrons strike it. The CCD camera then transforms these photons into electrical signals. A CCD chip has an array of photosensitive elements, in which electrical charge accumulates. Charge is transferred to a read out register, amplified and digitised to form an image. The DQE of a CCD camera is inferior to film at the electron energies typically used for imaging. The scintillator layer results in electron and thus photon scattering, causes a loss in sensitivity as well as introducing an accelerating voltage-dependent point spread function. Despite these problems, data collected on a CCD camera under appropriate imaging conditions can be processed to sub-nanometre resolution [60].

In the last few years, the use of DEDs has revolutionised biological TEM. Combining the benefits of a high DQE detector with the convenience of an electronic read out, DED’s are now the detector of choice for high-resolution EM [61]. MAPS detectors are currently the most widespread; they detect incident electrons directly as they pass through a semiconductor wafer ∼10 μm in depth, where they deposit energy. Some electrons are back scattered from the support matrix and pass through the semiconductor again, generating noise. As a result, high DQE DEDs are extensively ‘back thinned’, which involves removing as much of the support matrix as possible (to <50 μm). This dramatically reduces back-scatter, and thus noise, as most electrons pass right through the detector. Monte Carlo simulations show the thinner the substrate the less scattering is observed [62]. DEDs are available commercially from three manufacturers, FEI, Direct Electron and Gatan, all of which have a DQE profile better than film [59].

A major, additional benefit of DED’s is their ability to spread dose across multiple frames, creating a movie of the exposure. This advancement was made possible by the DED’s high frame rate. This is invaluable, as each frame can be aligned relative to each other, allowing for the correction of specimen movement (caused both by mechanical drift and beam induced movement) [63], [64], [65]. Structures at near-atomic resolution have been determined using all three manufacturers’ DEDs [16], [36], [66], [67].

### 4.3 Energy filters and phase plates

Low dose imaging means that cryo-EM produces images with poor SNR. To maximise the high-resolution information in images, they must be taken close to focus, where image contrast is minimal [12]. To combat this, two complementary technologies have been developed to increase SNR in images: phase plates and energy filters.

When imaging using electrons, a proportion of electrons that interact with the specimen are inelastically scattered, i.e. they deposit energy in the specimen. Their resulting lower energy, and thus longer wavelength, means they are focused in a different plane to elastically scattered electrons [12]. Inelastically scattered electrons can be considered a form of chromatic aberration, introducing noise. Energy filtering can prevent inelastically scattered electrons contributing to the recorded image. There are two energy filters currently available, the in-column Omega (Ω) filter and post-column Gatan energy filter (GIF) [58]. Both work on the principle that electrons of different energies/wavelengths can be deflected along different paths, meaning that only electrons that interact elastically (or not at all) with the specimen contribute to the image. This can dramatically improve the SNR. Energy filtering is particularly useful for collecting tilt series, as at high tilts the specimen becomes thicker in the direction of the beam, and the amount of inelastic scattering is proportional to thickness.

When imaging unstained biological specimens, there is little amplitude contrast [12]. However, this problem can be offset by defocussing the microscope, introducing phase contrast, albeit at the cost of a resolution-dependent loss of information. Phase plates have been developed to ameliorate this problem. They work by introducing an additional phase shift to the more strongly scattered components of the beam. Phase plates allow imaging at (or close to) focus, retaining information while generating sufficient contrast to visualise the specimen. While the concept has been in development for many years, the practical application of phase plates has only been realised in the last few years. There are two main types: Zernike and Volta potential.

The Zernike phase plate (ZPP) is a thin carbon film with a small (∼1 μm) hole, which is aligned so the unscattered beam passes through it [68]. Scattered electrons pass through the carbon film and undergo an approximate π/2 shift, relative to the unscattered beam. This causes the contrast transfer function (CTF) to switch from a sine to a cosine function, boosting S/N at low spatial frequencies and providing image contrast. ZPPs are commercially available in JEOL EMs and have been used in tomography experiments to beautiful effect [10]. However, images taken using a ZPP can suffer from fringing artefacts around high contrast features, owing to the sudden onset of the CTF [69]. ZPPs are also time consuming to align, and have a short, unpredictable lifetime (∼1 week), that is independent of use.

Volta potential phase plates (VPPP) use an ∼12 nm thick film of amorphous carbon heated to 200 °C to introduce the required phase shift [70]. The physics underlying this phenomenon remains unclear, but it is hypothesised to function by a beam-induced Volta potential [70]. This may arise from beam-induced surface modification of the carbon, together with a chemical equilibrium between the carbon surface and residual gases in the microscope column. VPPPs do not introduce fringing artefacts, and they are reported to be stable for months [70]. As VPPPs have been developed recently, they have not been extensively tested, but are now available commercially in FEI microscopes.

As both phase plate designs boost SNR at low spatial frequencies, they are particularly useful when performing electron tomography. For single particle work it remains to be seen how much benefit phase plates will yield. In principle however, so long as they efficiently transfer information at high-spatial frequencies, they may reduce the amount of data needed to generate a structure to the same resolution compared with a dataset collected with no phase plate [71], and may be beneficial for samples that are particularly small or hard to align [72].

### 4.4 Automated data collection

The SNR of cryo-EM images is very low and so many images of individual particles (>1000s) are usually needed to generate a structure using single particle processing methods [2]. The number of micrographs required is highly variable, and dependent on the specimen (for example, its size, its adoption of preferred orientations, and symmetry), the number of particles per micrograph, desired resolution, and levels of heterogeneity in a dataset. As a result, several software packages have been developed to automate data collection, and make more efficient use of microscope time. One commonly used package is FEI’s automated data collection software EPU, or ‘E Pluribus Unum’. Open-source programmes with similar functionality have been developed, such as Leginon [73], [74], TOM^2^ [75], [76] and SerialEM [77]. Software to allow the automated collection of tilt series has also been developed, including SerialEM [77], UCSF Tomo [78], Xplore3D (FEI) and Leginon [79]. Efforts are also underway to introduce semi-automated data processing pipelines for cryo-EM data [80].

### 4.5 Access to facilities

While recent advances in hardware are exciting, high-end microscopes, DEDs and associated equipment are expensive to purchase, operate and maintain. The MRC LMB, Cambridge UK estimates its running costs as £3000 per day, plus another £1000 on electricity [81]. The difficulty and expense of acquiring and maintaining state-of-the-art microscope facilities is becoming beyond the reach of many laboratories.

There are many benefits to centralised scientific equipment facilities, which have grown in number as equipment has grown in size, complexity and expense. To make high-end cryo-EM more accessible, some shared facilities are becoming available, such as The Howard Hughes Medical Institute (HHMI) at the Janelia Farm Campus, Virginia, USA, that is open to HHMI-funded investigators based elsewhere. In The Netherlands, NeCEN is an open access cryo-EM facility. In the UK, the electron Bio-Imaging Centre (eBIC) at the Diamond Light Source (Harwell, UK) is a national cryo-EM facility, funded by grants from the Wellcome Trust and UK Research Councils [82].

The successful, efficient operation of a full 24 h external user program on multiple high-end electron microscopes requires a facility that remains open, accessible and safe over the entire time period. The logistics and expense of this are beyond the capabilities of most universities. The efficient organisation of an external user program on multiple high-performance microscopes is a significant undertaking, which requires many support staff. Data storage and processing requirements also present considerable challenges and these are greatly facilitated by access to large-scale centralised data storage and computational infrastructure. Onsite and 24 h on-call availability of expert staff to support these capabilities is crucial to optimal operation. The funding and construction of EM facilities in the USA and Europe has facilitated access to high-end microscopes to all in the scientific community [82]. It is hoped that the siting of eBIC at Diamond Light Source will benefit from the extensive onsite expertise in automating equipment, sample handling, correlative microscopies along with the dedicated software and detector groups to enable significant scientific advances in these areas such as have been achieved recently in the macromolecular crystallography (MX) community [83].

## 5 Complementary methods

While EM can be a powerful tool to probe structure and function in its own right, increasingly EM is being used in conjunction with other methods, or techniques to bring additional dimensions into experiments. This includes the docking and sometimes refinement of existing high-resolution structures from X-ray crystallography or NMR into density maps [84]. Here, two emerging methods that are adding extra dimensions to EM structural studies, correlative light electron microscopy and methods for time resolved EM, are discussed.

### 5.1 Correlative-light electron microscopy

Cryo-electron tomograms of a cell can be highly challenging to interpret, how can you unambiguously identify features? Whilst in some cases morphological identifications can be made with high confidence, such as imaging mitochondria and the cytoskeleton, many features of tomograms cannot be identified purely based on morphology. In addition, EM is increasingly being used to study spatially and temporally rare events, such as endocytosis and virus entry into cells [85], [86]. As a result, methods have been developed to find the ‘needle in the haystack’, or a specific object of interest and to make functional interpretations.

Fluorescence microscopy in combination with fluorescent markers and cloneable fluorescent tags has lead to huge advances in cell biology [87], [88], [89]. Through the use of different fluorescent labels, the spatial and temporal location of many proteins or cellular components can be simultaneously tracked. There have been two main approaches to replicate this kind of functionality in EM, the generation of cloneable tags/markers of points of interest, and correlative light electron microscopy (CLEM).

The two key questions when designing a CLEM experiment are what fluorescence microscope (FM) and EM techniques to combine, and how to perform the correlations. Many different microscopy techniques have been combined and solutions for correlation determination developed, a limited selection of which will be discussed here (Table 3). Methods for correlating information from two or more microscopy techniques fall into two categories. The first are integrated microscopes, capable of two imaging modalities. As the sample does not move between instruments, the risk of damage and contamination is reduced, and an area of interest can be located in one imaging mode then imaged directly using another. One commercially available option is the FEI iCorr system, which integrates a FM into a Tecnai G^2^ microscope [90]. A 15 × 0.5 NA lens is integrated; one laser line is fitted allowing the imaging of GFP, AlexaFluor488 or other spectrally similar fluorophores. The FM sits within the vacuum of the EM and is mounted perpendicular to the electron beam of the TEM, meaning the grid is rotated by 90° to switch between imaging modes. While integrated dual mode microscopes are convenient, they can be prohibitively expensive.

The second category involves performing the imaging on two separate instruments, and a fiducial marker system to navigate to the region of interest in the second imaging mode. While correlations of this kind can be more difficult to implement, they can be less expensive and more flexible than integrated systems. FM of samples cryogenically fixed (cryo-FM) is now increasingly being used in conjunction with TEM. It has been demonstrated that fluorophores do still fluoresce under cryo-conditions, and in fact bleaching of fluorophores is slowed [91].

Several microscope stages have been designed to perform FM under cryogenic conditions on both upright and inverted optical systems, and a wide range of microscopy techniques can be performed under cryogenic conditions including confocal microscopy and z stack imaging [91], [92], [93]. Very low cost cryo-light microscopy stages (<$40 USD) have been developed from materials commonly found in hardware stores, although their functionality and stability is limited compared with commercial models [94]. The most commonly used commercial cryo-FM stages are the cryostage^2^ by FEI, developed with Baumeister and colleagues [95], and the Linkam cryostage (Linkam Scientific Instruments) in collaboration with Koster and colleagues, and Leica EM cryo CLEM developed with John Briggs.

**Fig. 5:**
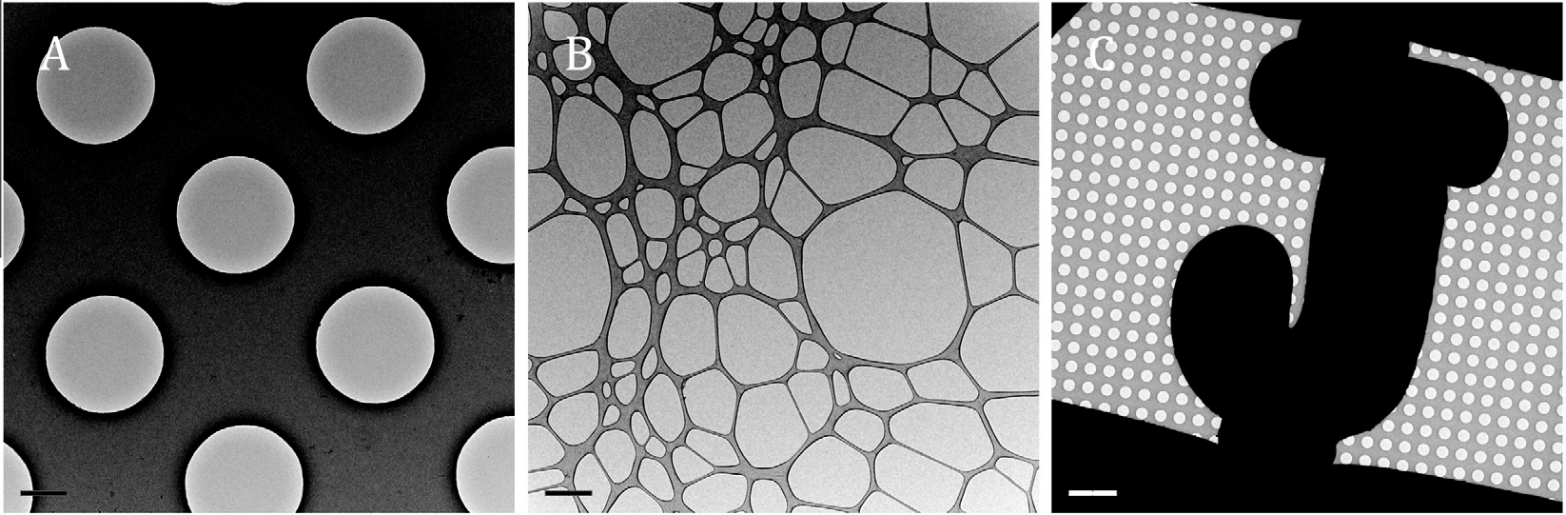
Examples of EM support films. A) Holey carbon, such as Quantifoil^®^ R2/2 (2 μm holes separated by 2 μm), Scale bar 1 μm. B) Lacy carbon film (irregular network of thin carbon), Scale bar 1 μm. C) Finder grid (here with Quantifoil^®^ R2/2 carbon), scale bar 10 μm.

With cryo-FM stages, markers across several orders of magnitude in scale must be used to navigate to the region of interest between the FM and TEM. Grids with large visual markers such as numbers, letters or symbols can be used for crude navigation (Fig. 5). Features within a grid square, such as regular Quantifoil carbon holes can also be used for crude correlations. For more accurate correlations in cryo-FM/EM, a combination of TetraSpek fluorescent microspheres and fluorescent electron dense microspheres can be used to perform accurate correlations to ∼60 nm [96].

Fluorescent, electron dense markers such as quantum dots (QD) are ideal for correlations. QDs are typically made of cadmium selenide or similar, with a shell such as zinc sulphide [97]. They can be covered with a polymer which allows a wide range of functionalisation, for example with streptavidin or Fab_2_ fragments, meaning they are also suitable for the labeling of proteins [97]. The fluorescence properties of QDs are ‘tuneable’ by their size, with smaller QDs typically fluorescing at shorter wavelengths [98]. In theory, this enables different QDs to be distinguishable in the TEM, however in practice this can be challenging, as their core is not very electron dense [98]. It has been shown in plastic embedded sections up to three QD sizes can be distinguished [97]. QDs are photostable and bright, making them ideal for fluorescence imaging techniques [99].

CLEM is a developing field, and in the coming years many more imaging techniques are likely to be combined. For example, stages are under development to allow super resolution imaging (e.g. PALM/STORM) of frozen-hydrated specimens [100]. Another interesting area that is likely to expand is using cryo-FM to guide cryo-FIB milling and cryo-ET, which has already been shown to be technically feasible [95]. By using this combination of techniques, a molecular resolution view of rare spatial or temporal events in thick regions of the cell such as the nucleus or perinuclear region can be generated [47].

### 5.2 Time-resolved EM

Macromolecular complexes can be dynamic, undergoing conformational changes under certain stimuli, such as binding to a ligand. These conformational changes can occur over variable timescales, from femtoseconds to hours [109]. Studying conformational changes which occur on short time scales can be especially challenging, and so sample preparation methods for cryo-EM have been adapted to enable time resolved analysis of macromolecular complexes down to <10 ms [110]. By plunge freezing specimens for cryo-EM, they are trapped within particular conformations and so by mixing two reactants, for example an enzyme and its substrate, a set time before vitrification, a temporal dimension can be incorporated into the experiment.

One of the most common approaches to achieving time resolution utilises a spraying technique, based on the early work by Unwin and Berriman, where one reactant is sprayed onto the grid and a second reactant sprayed on before freezing [111]. In an alternative approach, rapid mixing of the two reagents is performed, which are then sprayed onto a thin film that is rapidly vitrified. The fastest reported time resolution has been achieved using a monolithic micro-fabricated silicon device that incorporates a mixing mechanism, incubation channel and pneumatic sprayer in a single chip [110], [112]. It is capable of achieving mixing within 0.4 ms and a minimum reaction time of 9.4 ms [110]. Using this methodology, the dynamics of bridges associating the 30S and 50S bacterial ribosomal subunits was examined [110]. This future development is likely to greatly improve in the next decade with increased interest and development in the EM field. A complementary approach also under development is the mapping of continuous states, where defined integer states do not exist [113].

## 6 Computational considerations

For many biological specimens, collecting a good quality data set is only the start. Processing of cryo-EM data can represent a significant challenge. Here, data-storage and computational considerations are discussed. EM structures rely on averaging of thousands of molecular views after imaging in single particle processing, or the collection of many images as part of a tilt series in tomography experiments. Thus EM has always been inherently and unavoidably computationally expensive. While the recent advent of direct electron detecting cameras with their high frame rates has revolutionised cryo-EM structure determination, this comes at an additional, prodigious data storage and computational cost.

When using traditional CCD cameras, the data storage problem is significant but manageable. A 32-bit 4k × 4k MRC format image of the kind captured by a high-end CCD camera such as a Gatan US4000 is ∼64 Mb in size. If a 10,000 particle dataset is required and 100 particles are captured on each micrograph, this represents ∼6.4 Gb of raw image data. However, whilst a typical DED camera such as the FEI Falcon 2 may still be a 4k × 4k sensor, it operates at 17 Hz. A typical 2 s exposure on a microscope is actually captured as a 35 frame movie, which is ∼2.2 Gb in size. Larger datasets are also required to allow for high-resolution structure determination. Finally, to allow for higher resolution, the magnification of the images will likely be significantly higher to give a finer object sampling that can describe high-resolution features. This increase in magnification reduces the field of view, and thus captures fewer particles per micrograph. A typical dataset collected can easily exceed 5 Tb of data. Therefore, at the level of the individual dataset, DEDs increase the data storage requirements by 2–3 orders of magnitude. It should be noted however that a Gatan K2 summit detector can capture 8k × 8k images, at frame rates up to 40 Hz, which might add a further order of magnitude difference to this calculation.

At the level of the EM Facility, a 24-h unattended data collection run using automation software typically might capture ∼1800 such exposure movies, or ∼4 Tb of data. At 300 days of operation per year, this would represent 1.2 Petabytes of raw image data. This is increased further with downstream processing such as drift correction. Careful consideration therefore needs go into designing the data storage infrastructure to support such facilities, and the data retention policies they operate. Practically, this starts with the basic networking infrastructure. 10 Gbit Ethernet connections are a sensible minimum requirement for moving this scale of data, and 40 Gbit may be desirable. Modular data storage servers and file systems that can scale to multi-petabyte sizes are required, but careful decisions must be made about what is stored and for how long, because long-term archival of every byte generated will be prohibitively expensive at marginal benefit. A more sensible approach may be to keep all data, including movie frames, while a project is live, but then only storing motion-corrected averages long term. This would reduce the scale of raw data by ∼30–60-fold.

Providing the computational resources to support structure determination is a separate, significant challenge. The resources required scale rapidly with the physical size of the object and vary quite significantly between image processing packages. Using the commonly used RELION package as a reference, structures in the Ranson laboratory were routinely determined using single, multi-core servers based on both Intel and AMD processors. For example, a quad socket, 64-core, 256 Gb server was used to determine a structure in a 480 × 480 × 480 volume to 3.0 Å resolution, including movie processing [36]. However, for larger structures, more rapid structure determination and the determination of multiple structures simultaneously, we now routinely use large linux clusters. Clusters with both 10 Gbit and Infiniband interconnects appear to work extremely well, and large structures routinely use hundreds of cores. It should be noted that our experience suggests that refinement of the ‘average structure’ results in the generation of 2–5 Tb of data in addition to the raw data. For those without access to computers sufficiently powerful to carryout processing tasks, cloud computing services may represent a flexible and cost effective solution [114], although care needs to be taken that such solutions comply with local data protection policies.

## 7 Challenges and outlook

**Fig. 6:**
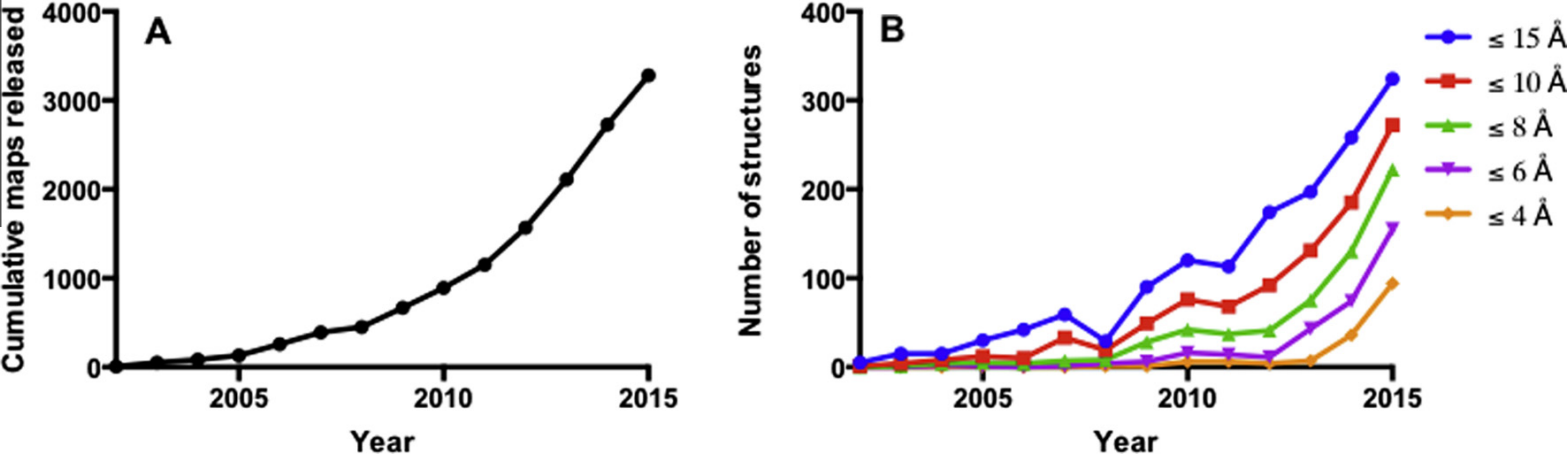
Number of structures in the EMDB. A) Cumulative map releases in the EMDB between 2002 and 2015. B) Map releases at given resolution levels between 2002 and 2015.

There is an emerging trend to integrate information from a range of different structural techniques, including X-ray crystallography and NMR. The Protein Data Bank (PDB) contains >100,000 structures elucidated by X-ray crystallography and >11,000 by NMR, including both multi-protein complexes and single-chain proteins and peptides. However, a broad range of biologically interesting proteins and protein complexes have resisted structural determination by these techniques, either because of difficulty in crystallisation, or because their size or dynamic properties currently preclude structure determination by NMR. By comparison, cryo-EM is a rapidly growing method for high-resolution structure determination. The Electron Microscopy Data Bank (EMDB) contains >2500 released density maps by single particle processing, helical reconstruction and electron diffraction; trends show the number of depositions is growing year on year, with many more high resolution structures being released (Fig. 6). However, issues remain with the validation and corroboration of reported resolution values. Unlike X-ray crystallography’s R-free value, there is currently no objective and straightforward quality criterion that is simple and easy to use. Even the reporting of density map resolution is subject to intense and ongoing debate and controversy.

While the emergence of cryo-EM and single particle techniques as a powerful tool for high resolution (<5 Å) structure determination is exciting and is changing our understanding of a range of macromolecular complexes, structure determination by EM methods to lower resolution can also be incredibly insightful. Cryo-electron tomography is revealing structural properties of complexes, sometimes *in situ*, at 5–10 nm range, while subtomogram averaging is allowing structures better than 10 Å to be obtained [115]. For density maps ∼8–20 Å, crystal or NMR structures of individual complex components can be fitted into electron density maps to yield biological insight [116], [117]. At even lower resolutions, negative stain EM can be used to gain insight into conformational flexibility or location of complex subunits. In conclusion, EM is a highly flexible technique, able to offer biological insight into a range of different biological questions at different size and resolution scales, and in combination with complementary structure determining and imaging techniques, represents a powerful tool in a new age of integrative structural biology.

## Figures and Tables

**Table 1:** Typical uses of common grid preparation methods.

Grid preparation method	Typical samples	Typical equipment	Typical results	Auxiliary equipment	Additional considerations
Negative staining visualisation	Macromolecular complexes (>50 kDa), organelles, prokaryotic cells	EM with tungsten filament	Visualisation of specimen with high contrast	– Glow discharge unit/UV lamp for treating carbon surfaces	– Different stains such as uranyl acetate or ammonium molybdate may be optimal for different specimens – Some sample buffers may cause problems, including presence of detergent, phosphate, reducing agent or glycerol – Sample may become (sometimes severely) deformed by the stain – -Sample may adopt preferred orientations on the carbon – Optimised stain depth is important for obtaining the best results
Negative staining reconstruction	Macromolecular complexes that appear reasonably homogenous	EM with LaB6 filament, CCD detector	Resolution limited by the grain size of specimen, typically to ∼20 Å at best	– Software and computer hardware to process data
Cryogenic visualisation	Macromolecular complexes (>150 kDa, liposomes, organelles, prokaryotic and eukaryotic cells	EM with LaB6 or FEG filament, CCD or DED and cryo-holder and sample transfer station	Visualisation of specimen with low contrast	– Software and computer hardware to process data	– Specimen contrast is low compared with negatively stained samples – Sample buffer components can reduce specimen contrast, such as glycerol, sucrose and detergent – Vitrification process may require optimisation for best results, including optimising blotting conditions and support films. When a thin continuous carbon support film is used, sample may adopt preferred orientations on the carbon – Samples smaller than ∼ 500 kDa can be very challenging to visualise – Typically, samples must be 10–50× more concentrated than used in negative stain visualisation to achieve similar particle distributions
Cryogenic reconstruction (single particle)	Macromolecular complexes (>150 kDa	As cryogenic visualisation, DED preferred	Depending on the specimen, reconstructions of ∼3–20 Å	– Time resolved EM where applicable	– Support film can dramatically influence particle distribution. A continuous carbon film can aid particle distribution, but this can introduce preferred particle orientations and noise into the image
Cryogenic reconstruction (tomography)	Organelles, prokaryotic cells, thin edge or lamellar of eukaryotic cells	As cryogenic visualisation, DED preferred, energy filters and phase plates can be of great benefit	Resolution of tomograms < 10 nm, subtomogram averaging can produce reconstructions of >10 Å	– CLEM – FIB milling or HPF and sectioning, where sample is too thick for direct visualisation	– Use support films with 200 mesh size to allow high tilts – Additional of gold fiducial markers may be helpful to aid alignment of tilt series images

**Table 2:** Comparison of commercially available plunge-freezing devices.

Model	Humidity control in chamber	Temperature control in chamber	Automatic control of ethane temp	Blotting modality	Additional information
Leica EM GP	✓	✓	✓	Single sided	Optional stereomicroscope to monitor blotting
Gatan Cryo-plunge 3	✕ (Chamber set at 98%)	✕ (Operates at ambient temperature)	✓	Single and double sided	Removable humidity chamber
FEI Vitrobot	✓	✓	✕	Double sided	Currently the most common plunge freezing device.

**Table 3:** Topics of study and microscopy techniques combined in CLEM.

Study	Imaging technique 1	Imaging technique 2	Reference
Intracellular dynamics	Live cell FM	Immunogold labeling resin embedded cell sections	[101]
GFP labeled proteins	Live cell FM	ET of resin embedded sections (di-aminobenzidine photo conversion using GFP bleaching)	[102]
Endosome dynamics	Live cell FM	Ultrathin cryo-sections with immunogold labeling	[103]
Endocytosis in yeast	FM on resin embedded sections	ET on resin embedded sections	[104]
HIV-1 infection	Time lapse live cell FM/cryo-FM	Cryo-ET at thin edge of whole HeLa cells	[105]
Mitochondria	Cryo-FM	Cryo-ET at the thin edge of HUVEC cells	[106]
*Herpesviridae* proteins	Cryo-FM (in column)	Cryo-soft X-ray microscopy	[107]
Endosomes	Cryo-FM (Linkam cryo stage)	Cryo-soft X-ray microscopy	[108]
Adenovirus particles	CryoFM (FEICryostage^2^)	Cryo-EM	[96]
